# Inactivation of helminth eggs in an electro-Fenton reactor: Towards full electrochemical disinfection of human waste using activated carbon

**DOI:** 10.1016/j.chemosphere.2020.126260

**Published:** 2020-07

**Authors:** Irma Robles, Emmanuel Becerra, J.A. Barrios, C. Maya, B. Jiménez, Francisco J. Rodríguez-Valadez, Fernando Rivera, Josué D. García-Espinoza, Luis A. Godínez

**Affiliations:** a Centro de Investigación y Desarrollo Tecnológico en Electroquímica S.C., Parque Tecnológico Querétaro, Sanfandila, 76703, Pedro Escobedo, Querétaro, Mexico; b Instituto de Ingeniería, Universidad Nacional Autónoma de México, 04510, CDMX, Mexico

**Keywords:** Helminth egg inactivation, Electro-Fenton, Electrochemical disinfection, Advanced oxidation processes, Human wastewater treatment

## Abstract

The disinfection of helminth eggs and *Escherichia coli* contaminated aqueous solutions, was studied using an electro-Fenton reactor equipped with a polarized activated carbon (AC) packed bed and two chambers loaded with cation exchange resins. Experiments using different arrangements and operation conditions, revealed that effective elimination of *Escherichia coli* takes place in all electrochemical disinfection tests. For the more resistant helminth eggs however, adsorption, electro-oxidation and electro-Fenton experiments showed retention within the reactor and pathogen inactivation values of 0, 16, and 25%, respectively. Using helminth eggs concentration data in different sections of the reactor, optical microscopy analysis and an exploratory computer simulation, differences in the disinfection performance were explained and new recirculation and flow direction and polarization switching operation schemes were defined. The corresponding experiments revealed that the effective coupling between adsorption and electro-Fenton phenomena, all along the AC packed bed compartment, results in 100% inactivation of helminth eggs.

## Highlights

• An activated carbon bed was used in an electro-Fenton reactor for disinfection purposes.
• *E. Coli* elimination was achieved in electrochemical experiments.
• An alternating electro-Fenton process is capable of fully inactivating *helminth eggs.*
• The approach points to the development of competitive electro-Fenton disinfection technology.

## Graphical abstract

**Figure:**
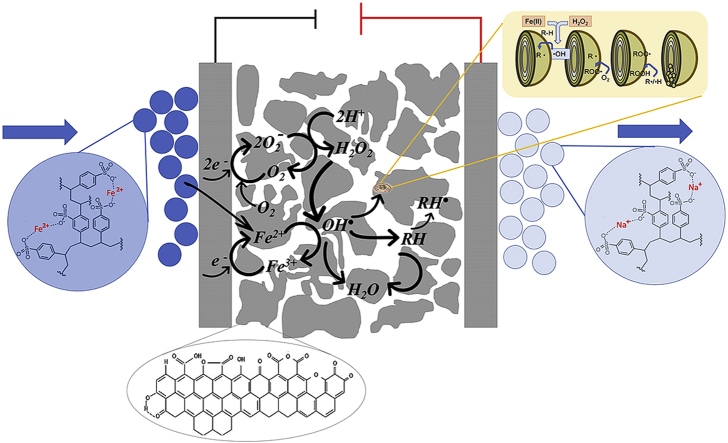

## 1 Introduction

Parasitic infections caused by helminths are considered neglected tropical diseases and the main cause is commonly related to inadequate sanitation. These parasites are transmitted through their egg, which remain a challenge for wastewater treatment facilities as they are highly resistant to conventional processes (Jimenez, 2007; Oh et al., 2016). Moreover, the presence of these pathogens in wastewater, seriously limits its reuse and constitutes an important health problem around the world. It is for instance, estimated that around 2600 million people worldwide are infected with helminths (Jiménez et al., 2016).

Helminth eggs (*HE)* are the infective stage of several intestinal worms and although several types of *HE* have been identified, all of them are characterized by a strong protection membrane made of an internal lipoidal shell, an intermediate quitinose layer and a proteic external shell (Ayres et al., 1992; de Silva et al., 1997). Due to the membrane resistant features of *HE*, the most popular methods to remove them from wastewater are based on a separation process (sedimentation, coagulation-flocculation and filtration) rather than on a degradation or inactivation approach (Muna, 2004; Oh et al., 2016).

In this way, it is clear that for the development of better and more environmentally friendly wastewater treatment systems, strong oxidation methods to destroy the membrane of *HE* are necessary and it is in this context, that recent studies have shown promising results by approaching the problem through advanced oxidation processes (AOPs) (Barrios et al., 2015).

AOPs are characterized by the generation and use of the OH radical species (•OH) which is a powerful oxidant characterized by a redox potential that is higher than that of hypochlorite, permanganate and H_2_O_2_ (2.8 V *vs* HRE (Hydrogen Reference Electrode)) and therefore, capable of oxidizing the protecting membrane of *HE*. Unless the pollutant is an halogenated compound, the oxidation by-products in AOPs are non-halogenated and since the •OH radical is a short-lived species, the effluent is usually harmless. Among the different AOPs that have been used to treat *HE* containing effluents, studies employing ozone, photocatalysis, electro-oxidation, Fenton and electro-Fenton processes, have been reported.

Ozone for instance, has been used to achieve above 90% of *HE* inactivation either by itself (in relatively high concentrations) (Orta-de Velásquez et al., 2002; Orta De Velásquez et al., 2004; Zamudio-Pérez et al., 2014), or combined with other treatment approaches such as active chloride (Corona-Vasquez et al., 2002) or microwave radiation (Mun et al., 2009). Fenton mixtures (H_2_O_2_ and Fe(II)) that readily produce •OH radicals, have also been successfully used (between 60 and 80% *HE* inactivation) (Escobar et al., 2014), testing different operation parameters and concentration ratios (Escobar-Megchún et al., 2014; Morales-Pérez et al., 2016) and in combination with other processes so that in the best case scenario, synergistic effects can be found. (Bandala et al., 2012, 2011; Ramírez Zamora, 2006).

While electromagnetic radiation, particularly UV light, has also been employed with TiO_2_, porphyrins or H_2_O_2_ to successfully photo-assist *HE* oxidation processes (pathogen inactivation values usually fall between 45 and 80%), (Alouini and Jemli, 2001; Garcia et al., 2008; Guadagnini et al., 2013; Leal et al., 2006), electrochemical oxidation approaches, have exploited the properties of electro-generated hypochlorite (Talekar et al., 2018).

Although these reports clearly show that the potential of the •OH radical to provide a solution for *HE* contaminated wastewater, there are very few reports that achieve the necessary complete *HE* inactivation of this pathogen and in the cases in which the efficiency is high, the cost is a limiting factor for the development of a useful and competitive technology.

On the basis of recent reports on the development of electro-Fenton processes for the •OH radical based oxidation of pollutants in waste water (Bañuelos et al., 2013; Esquivel et al., 2009; Fernández et al., 2018; Robles et al., 2016), we are presenting in this report the results of experiments in which different experimental conditions were surveyed to achieve complete *HE* inactivation using an electrochemical reactor that works with inexpensive materials and small energy requirements. It is hoped that this approach could constitute the basis of a technically and economically viable technology for the treatment of human wastewater contaminated with *E. coli* and *HE*.

## 2 Material and methods

### 2.1 Materials

All the solutions employed in this work were prepared using the synthetic formulation for domestic wastewater reported by Castro (2014). Once prepared, 1 L of each one of these solutions were contaminated with 203 ± 3 helminth egg units plus *Escherichia coli* strain (ATCC® 700078™) to a density of 3.0 × 10^8^ CFU (Colony Forming Units) per mL; this density is typical of municipal wastewater according to Jimenez-Cisneros et al. (Jiménez-Cisneros et al., 2001).

The *Escherichia coli* was previously inoculated, cultivated and isolated on *McConkey* agar (DIFCO brand) at 35 °C for 24 h. Subsequently, it was re-suspended in saline solution (NaCl at 0.85%), thus obtaining a comparable turbidity to the tube number one on the McFarland scale. Then, an optical evaluation allowed the approximate concentration of the culture to be determined by comparing the turbidity of the liquid medium with a standard that represented a suspension of a known number of bacteria. On the other hand, the concentrated stock solution of different species of *HE*, was obtained from infected feces, adult female dissection, as well as wastewater and sludge samples. The identified species included *Ascaris lumbricoides, Trichuris trichiura, Toxocara canis, Hymenolepis nana, Hymenolepis diminuta, Taenia solium,* and *Hookworms*.

**Fig. 1:**
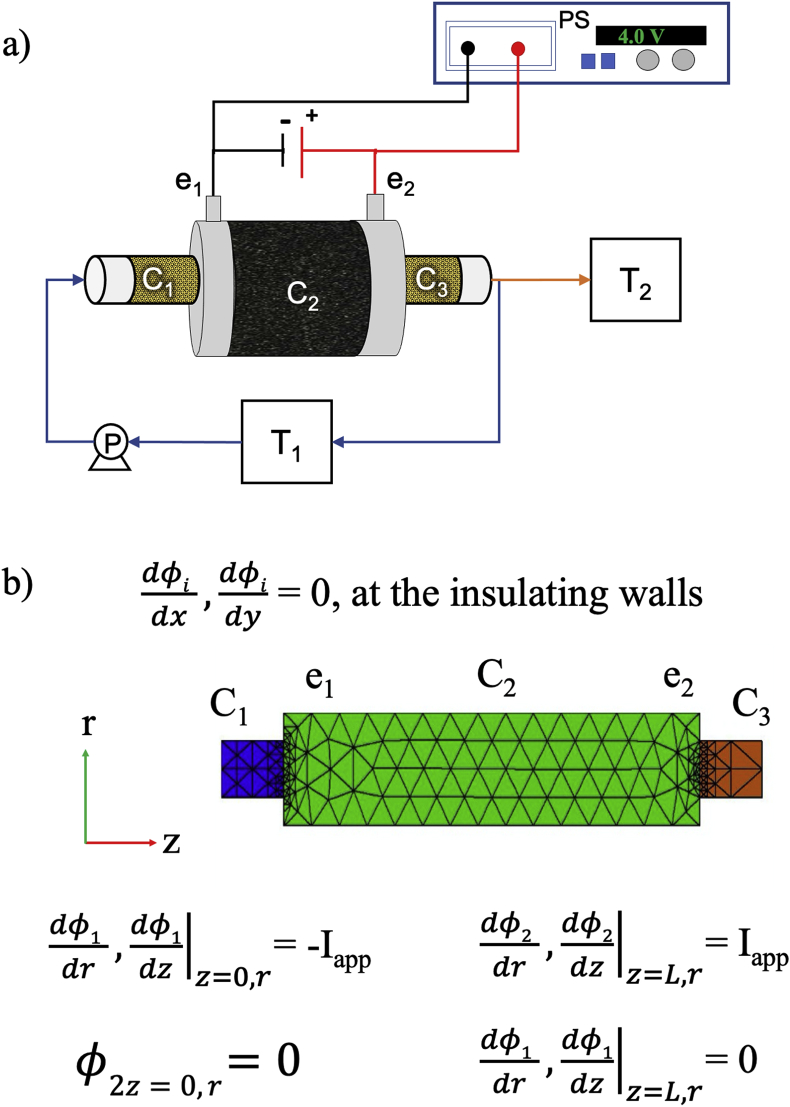
a) Schematic diagram of the experimental set-up and the components of the: (C_1_-C_3_) electrochemical reactor, (PS) power source, (P) peristaltic pump. T_1_ and T_2_ correspond to the feeding and the receiving tanks, respectively. b) Balance Equations and boundary conditions for mass, and energy balance for the electro-Fenton reactor under study.

After treatment, samples were taken from different parts of the reactor (see Fig. 1a) to evaluate inactivation of *E. coli* and *HE* using Mexican standard regulations NMXAA-102-SEMARNAT-2006 and NMX-AA-113-SCFI-2012, respectively.

*Escherichia coli* (*E. coli*) determination was carried out by filtering samples through sterile Millipore systems with a sterile cellulose acetate membrane and a gridded surface. Seeding and inoculation of *E. coli* were performed on Petri dishes with m-FC agar (DIFCO brand). Once the selected serial dilutions were filtered, Petri dishes with membranes were incubated in a water bath at 44.5 °C for 24 h. Following this, blue colonies, indicating *E. coli*, were counted.

Recovery and quantification of *HE* on the other hand, were performed by filtering samples through 170 and 20 μm sieves to retain particles within the range of inoculated *HE* (25–150 μm). Subsequently centrifugation at 2500 rpm was carried out, using a 1.3 density saturated solution of ZnSO_4_. In order to obtain a preliminary count of *HE* (viable and non-viable), staining of the pellet using a trypan blue assay was performed. *HE* quantification was performed by direct observation on a Sedgewick Rafter camera under 10× magnification with a Carl Zeiss XA optical microscope. Stained eggs were considered non-viable and unstained eggs, viable. To confirm viability results obtained by staining, eggs were recovered. Centrifugation in a centrifuge IEC, model HN-SII and filtering through a 20 μm pore size sieve (model US3-850S, DUAL MFG. CO. INC) were carried out followed by further incubation under acidic conditions using acid-alcohol solution (70%–30%) for 20 days at a temperature of 28 °C. Viability confirmation was completed through direct observation using an optical microscope.

CG8-C cation exchange resin, carbon cloths and activated carbon (AC) were obtained from Resintech (Mexico), ROOE (Mexico), and Clarimex (Mexico), respectively. Prior to setting up the electrochemical reactor, the resins and the carbon materials were cleaned and pre-treated as previously described (Zárate-Guzmán et al., 2018).

### 2.2 Electrochemical experiments

Based a previous report by our group (Fernández et al., 2018), a laboratory scale electro-Fenton reactor with a volume of 300 cm^3^, was set-up as shown in Fig. 1a. In this way, while two carbon cloth electrodes were used to polarize an activated carbon (AC) bed (0.54 g cm^−3^) (Zárate-Guzmán et al., 2018) located in the middle part of the reactor (C_2_), two compartments (C_1_ and C_3_), loaded with 2 g of a cation exchange resin, were connected to each side of the C_2_ compartment using stainless steel screws. Each one of these two compartments had either a connection to the outlet or the inlet tubing so that the contaminated solution could be pumped across the cylindrical reactor. For EF experiments, it is important to point out that while the inlet compartment, C_1_, is filled with resin previously loaded with Fe(II) and H^+^, the outlet compartment, C_3_, contains a cation exchange polymeric resin that was fully exchanged with Na^+^ cations. Fig. 1a also shows a power source (PS, Novak Technologies) that feeds the reactor with the required energy, and an air pump that is used to keep the wastewater solution saturated with air. In all experiments, the flow rate (14 mL min^−1^) as well as the treated wastewater volume (1 L) were the same.

As it will be described and discussed in the corresponding sections, three different sets of experiments were carried out using the reactor set-up shown in Fig. 1a. While in the first set of experiments (ADS, EO and EF) the contaminated solution (T_1_) was pumped through the reactor and the effluent was collected in a separate container (T_2_), in the second set of experiments (EF-R) the effluent coming out from the reactor was mixed back with the influent, so that the feeding and receiving container was the same (T_1_). Finally, and as opposed to the previously described experiments in which the treatment was continuous and lasted 72 min, in the third set of experiments, adsorption and electrochemical effects were sequentially applied and the flow direction and polarization were changed. In this way, while the wastewater solution in this process is sequentially pumped and polarized in 15 and 20 min stages (see Table 1), the electric field as well as the flow direction are sequentially switched (García-Espinoza et al., 2019).

**Table 1:** Operation conditions for the sequential adsorption/polarization stages in the EF-RPI process.

Stage	Time (min)	Flow Direction	Polarization, e_1_---e_2_
Adsorption, Fe and H^+^ introduction into C_2_	15	→	
Electro-Fenton	20		(−)---(+)
Adsorption, Fe and H^+^ introduction into C_2_	15	←	
Electro-Fenton	20		(+)---(−)
Adsorption, Fe and H^+^ introduction into C_2_	15	→	
Electro-Fenton	20		(−)---(+)
Adsorption, Fe and H^+^ introduction into C_2_	15	←	
Electro-Fenton	20		(+)---(−)
Adsorption, Fe and H^+^ introduction into C_2_	15	→	

Cyclic voltammetry experiments on the other hand, were carried out at room temperature using a BAS potentiostat and the reactor shown in Fig. 1a. While the working and counter electrodes consisted on the two carbon cloth substrates, the reference consisted on an Ag|AgCl electrode positioned close to the cathode (working) electrode.

### 2.3 Simulation of the reactor’s adsorption and electric potential distributions

The processes that will be discussed in the corresponding section, consider a preliminary pollutant adsorption stage and when is the case, the electrochemical activity of the different regions at the porous-type electrode reactor (2D subdomain is shown in Fig. 1b). In order to better understand the contribution of each one of the processes that take place within the reactor, a simulation of the adsorption and electric potential distribution was carried out. The mathematical model considers that for a porous electrode, electro neutrality between the electrolytic and the electrode phases prevails and hence, the divergence of the total current density must be zero.

(1) $$\nabla \cdot i_{1}+\nabla \cdot i_{2}=0$$

In equation (1), *i*_*1*_ corresponds to the local current density in a given point of the carbonaceous electrode and *i*_*2*_ is the current density at electrolytic phase. Considering a cylindrical coordinate system, the charge balance for each phase could be described by the 2D Laplace equation:

(2) $$\nabla \cdot i_{1}=-k_{mat}\nabla^{2}\varphi_{1}=-k_{mat}\left(\frac{1}{r}\frac{\partial }{\partial r}r\frac{\partial \varphi_{1}}{\partial r}+\frac{\partial^{2}\emptyset_{1}}{\partial z^{2}}\right)$$

(3) $$\nabla \cdot i_{2}=-k_{mat}\left(\frac{1}{r}\frac{\partial }{\partial r}r\frac{\partial \varphi_{2}}{\partial r}+\frac{\partial^{2}\emptyset_{2}}{\partial z^{2}}\right)$$

By combining equations (2), (3), (1), and considering Butler-Volmer type kinetics and non-uniform reaction rates, the expression to determine the potential distribution across the AC packed bed in C_2_, is:

(4) $$-k_{mat}\left(\frac{1}{r}\frac{\partial }{\partial r}r\frac{\partial \varphi_{2}}{\partial r}+\frac{\partial^{2}\emptyset_{2}}{\partial z^{2}}\right)=a\left[i_{0a}exp\left(\frac{\alpha_{a}F\left(\varphi_{1}-\varphi_{2}-E_{ocp}\right)}{RT}\right)-i_{0c}exp\left(\frac{-\alpha_{c}F\left(\varphi_{1}-\varphi_{2}-E_{ocp}\right)}{RT}\right)\right]$$

The boundary conditions that are used to calculate the potential and current distributions are schematized in Fig. 1b.

In the absence of polarization however, the adsorption of *HE* was the only process considered and mass balances in the AC packed bed media could be defined in terms of axial and radial flow dispersion coefficients, so that the effect of hydraulics inside the reactor can be described as:

(5) $$D_{ax}\frac{\partial^{2}C_{i}}{\partial z^{2}}+D_{r}\left(\frac{1}{r}\frac{\partial }{\partial r}r\frac{\partial C_{i}}{\partial r}\right)-\frac{Ueff}{\varepsilon }\frac{\partial C_{i}}{\partial z}-R_{i}=\frac{\partial C_{i}}{\partial t}$$

The parameters used for the model and its numerical solving procedure, are summarized in Table SM-1 (supplementary material).

The numerical solutions of the mathematical models for secondary potential distribution and mass balances using the boundary conditions shown in Fig. 1b, (which were the values used for adsorption and for the thermodynamics and kinetics of electron transfer in Table SM-1) were obtained using the *FlexPDE* software, 7.13/W64 professional version. The commercial software was used to solve partial differential equations by means of the finite element method. Different step and mesh sizes were tested, and the model response was considered to be independent once the number of elements was equal or larger than 12000.

It is also important to note that although electric potential and active species concentrations were solved simultaneously for the reactor in Fig. 1b, only the plots of *HE* distribution are shown.

## 3 Results and discussion

### 3.1 Adsorption (ADS) and electro-oxidation (EO) disinfection processes

Following a previous report (Fernández et al., 2018), the electrochemical reactor shown in Fig. 1a was built and used to study the disinfection of pathogen contaminated solutions. In this way, 1 L of synthetic wastewater in T_1_ (containing *HE* (203 ± 3) plus *E. coli* (ATCC 700078), 3.0 × 10^8^ CFU per mL) was fed into the reactor at a rate of 14 mL min^−1^ using a pump (P) and discharged in T_2_.

For the experiments described in this section, the reactor in Fig. 1a contained Na^+^ loaded cation exchange resins in sections C_1_ and C_3_, and an AC packed bed (Zárate-Guzmán et al., 2018) in compartment C_2_. In this way, while the adsorption operation mode (ADS) was carried out in the absence of applied potential, electro-oxidation experiments (EO) were performed imposing a 4 V potential difference between e_1_ and e_2_ (see Fig. 1a).

**Fig. 2:**
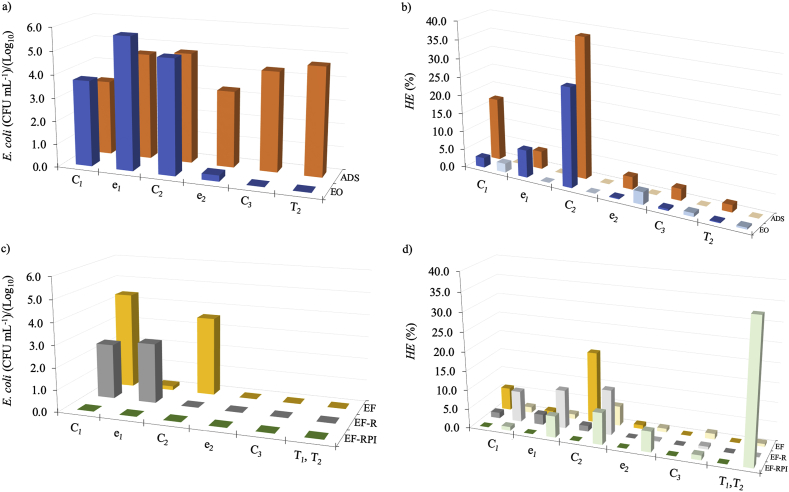
Concentration of *Escherichia coli* ((a) and (c)) and fraction of viable (V) and non-viable (NV) *helminth eggs* ((b) and (d)) in the different reactor compartments using ADS and EO ((a) and (b)) and EF, EF-R and EF-RPI ((c) and (d)) treatments. Dark and light colors represent viable and non-viable *HE (%)*, respectively. Initial concentrations for *E. coli* and *HE* correspond to log 10 8.48 CFU mL^−1^ and 203 L^−1^, respectively. (For interpretation of the references to color in this figure legend, the reader is referred to the Web version of this article.)

The results for ADS and EO in terms of the presence of *E. coli* in the reactor after the experiments, can be observed in Fig. 2a. Inspection of the corresponding data for ADS shows that the distribution of the pathogen along the reactor was fairly homogeneous since the concentration difference that was observed in the different regions of the reactor, was in all cases about 1 Log_10_ units. The adsorption process therefore, reflects a retention effect that is expected to fade away on time upon pathogen saturation of the different zones of the reactor.

The data for EO in Fig. 2a shows on the other hand that *E. coli* disinfection was highly effective, reaching values close to 100% in the treated water. In fact, *E. coli* concentrations for EO were found to be very small not only in the effluent (T_2_) but also on the C_3_ resin compartment and in the section of the reactor where the electrochemical oxidation stage of the treatment process takes place, i.e., the anodic carbon cloth electrode, e_2_. This observation is consistent with previous reports (Fisher and Nelson, 2014; Garkusheva et al., 2017; Rodríguez-Chueca et al., 2012) in which it is pointed out that electrochemical oxidation processes, are fairly efficient to disinfect *E. coli* containing wastewater.

*HE* on the other hand, constitutes a more challenging test since its protecting membrane is far more resistant than that of *E. coli.* As can be seen in the corresponding experimental data shown in Fig. 2b, the simplest arrangement, ADS, results in the effective removal of the pathogen from solution (90%). Consistent with previous observations however, this effect is limited to a physical separation of the pathogen from the aqueous effluent since *HE* inactivation could not be detected in any of the sections of the reactor (Fig. 2b). As it is the case for *E. coli*, ADS implies that after the adsorbent substrate is saturated, pathogen adsorption will cease and the removal effect will be over.

Applying a 4 V electric potential difference across the AC packed bed on the other hand, results in the coupling of adsorption effects with the electrochemical induced attack of the pathogen agent. Inspection of the corresponding results in Fig. 2b, reveals that the EO treatment produces the inactivation of about 16% of *HE* of the influent solution. Analysis of the distribution of inactivated pathogens in the different sections of the reactor (see Fig. 2b) also reveals that as expected, the inactivated fraction of the total number of *HE* pathogens varies along the different sections of the reactor. In fact, inspection of the distribution of non-viable *HE* shows that the applied potential promotes a modest *HE* inactivation process that preferentially takes place in the vicinity of the electrodes.

### 3.2 Electro-Fenton (EF) disinfection process

The next step consisted on using the reactor in Fig. 1a to carry out electro-Fenton disinfection tests. As described in the previous report by (Fernández et al., 2018), an arrangement of three compartments in series results in an electro-Fenton process for neutral, iron-free solutions, in which three sequential steps occur as the effluent goes through the reactor. In this way, an Fe(II) + acid loaded cation exchange resin (Ramírez et al., 2010) located in C_1_ provides the two chemical species that are necessary for the Fenton reaction as the pathogen contaminated aqueous solution washes out the material of the first compartment (C_1_) and takes it to C_2_. In the middle region of the reactor (second compartment, C_2_), a polarized activated carbon (AC) bed simultaneously works as an adsorbent substrate of pollutant agents, as well as a 3D-type cathode for oxygen reduction. As can be seen in equation (6), the product of this electrochemical reaction is H_2_O_2_ which, as described by equation (7), readily reacts with Fe(II) to produce the •OH radical species (Parsons (ed), 2004).

(6) $$O_{2}+2H^{+}+2e^{-}\to H_{2}O_{2}$$

(7) $$Fe^{2+}+H_{2}O_{2}\to HO^{•}+OH^{-}+Fe^{3+}$$

Since •OH is a powerful oxidant, the hypothesis is that •OH will readily react with any pollutant that is adsorbed on the carbon electrode surface, resulting in a process in which fast pathogen oxidation (Bañuelos et al., 2013) is coupled to an effective regeneration process of the adsorbent surface (García-Rodríguez et al., 2015).

Finally, as the effluent leaves C_2_, the oxidized by-products in solution reach the third compartment, C_3_, where Fe(II), Fe(III) and H^+^ are partially retained in the cationic exchange resin, rendering in T_2_ disinfected Fe-free neutral water.

**Fig. 3:**
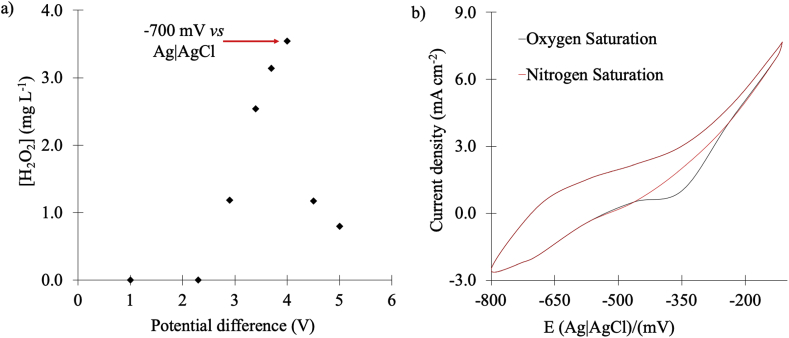
(a) Concentration of electrochemically produced H_2_O_2_*vs* the applied potential difference across C_2_ in the reactor shown in Fig. 1a. Flow rate 14 mL min^−1^. (b) Cyclic Voltammetry responses at room temperature of a carbon cloth electrode in O_2_ (black) and N_2_ (red) saturated, 0.05 M Na_2_SO_4_ solution, using a Ag|AgCl reference electrode and a scan rate of 50 mV s^−1^. (For interpretation of the references to color in this figure legend, the reader is referred to the Web version of this article.)

In order to promote the electro-Fenton induced reactions described in equation (6) however, it was necessary to first determine the potential difference that had to be applied between the carbon cloth electrodes positioned between C_1_ and C_2_, and C_2_ and C_3_. To do this, an oxygen saturated electrolytic solution was pumped across a reactor characterized by empty C_1_ and C_3_ compartments and a C_2_ middle section packed with AC (Zárate-Guzmán et al., 2018). Then, different polarization voltages were imposed between the carbon cloth electrode contacts and the concentration of H_2_O_2_ in the effluent was measured as described in (Bañuelos et al., 2014). In Fig. 3a the data of these experiments is shown, revealing that while the concentration of electro-generated H_2_O_2_ gradually increased from 2.2 to 4.0 V, at higher applied potential values a drastic decrease of the oxidant concentration takes place. The change observed at 4 V reflects the onset at which electrochemical peroxide degradation, or a 4 e^−^ reduction process of dissolved oxygen, occurs (Peralta-Hernández and Godínez, 2014).

This interpretation was confirmed by the Cyclic Voltammetry (CV) responses of the system under N_2_ and O_2_ saturation conditions. Inspection of the corresponding voltammetry data in Fig. 3b shows that the potential onset for the 2 e^−^ reduction of oxygen is observed at about −250 mV (measured by positioning a Ag|AgCl reference electrode close to the carbon cloth working as a cathode) and that at −700 mV, which corresponds to a 4 V potential difference between the two carbon cloths in the electro-Fenton reactor, a stable production of H_2_O_2_ takes place.

Once the conditions for the process were stablished, EF disinfection tests were carried out. The results for *E. coli* inactivation using the EF operation mode in the reactor in Fig. 1a are shown in Fig. 2c. Inspection of the corresponding data shows that as expected, *E. coli* inactivation was very effective since only a small amount of this pathogen could be assessed in the carbon cloth anode, e_2_, and no pathogen could be detected in the third compartment as well as in the effluent, T_2_. It is also interesting to see that, as opposed to what was observed for EO, the concentration of *E. coli* in the cathodic carbon cloth electrode region, e_1_, is substantially smaller than that observed in the AC compartment (C_2_). In fact, the two complementary effects of the cathodic and anodic disinfection reactions can be observed by comparing the *E. coli* concentration distributions for EO and EF in Fig. 2a and c. In this way, since the oxidation environment is limited to the anodic part of the reactor in EO, the observation of a smaller concentration of *E. coli* in e_1_ for EF, confirms that in the electro-Fenton process, the generation of the powerful •OH radical species takes place in the cathodic part of the reactor; *i.e.*, in the region in which the reactions described by equations (6), (7) take place.

**Fig. 4:**
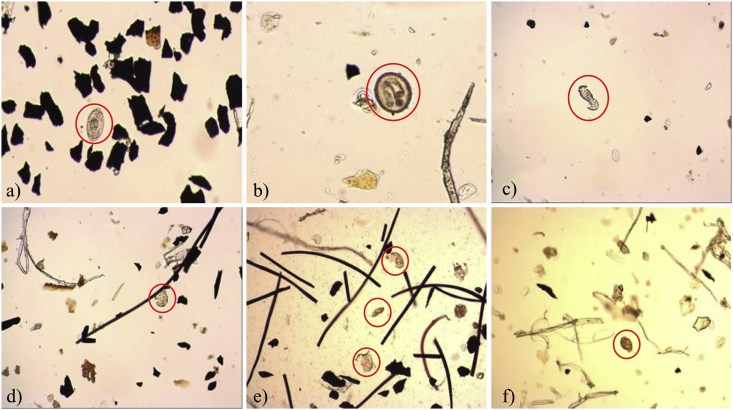
Optical images of *HE* for the different working conditions under study. a) *HE* (*Hymenolepis nana*) from AC (C_2_) after ADS and b) Viable *Toxocara canis* egg from e_1_ after ADS, show viable *HE* images. c) and d) damaged eggs of various helminth species after EF process; e) eggshell remains after EF-RPI; and f) *Ascaris lumbricoide* after EF-RPI.

Experimental results of EF experiments for the more resistant *HE* pathogen are shown in Fig. 2d. Inspection of the corresponding data shows that, as it was previously observed for EO, most of the *HE* pathogen is retained along the reactor in the AC compartment, C_2_. The inactivation of *HE* using the EF approach however, reaches a value close to 25% (see Fig. 2d); about 9% higher than that observed for the EO process. It is also interesting to note that the small amounts of *HE* found in C_3_ and in the effluent, T_2_, all correspond to inactive pathogen agents. The extent of the •OH promoted *HE* damage, was confirmed from images taken from samples obtained in different parts of the reactor. As shown in Fig. 4c and d, and contrary to the viable *HE* images shown in Fig. 4a and b corresponding to the ADS process, egg damage was detected when the •OH radical produced in the EF approach is employed.

### 3.3 Optimizing disinfection trough re-circulation

Since we believed that the good results obtained for EF were related to adsorption of the pathogen followed by electro-Fenton induced disinfection of the adsorbed species, we decided to re-circulate the solution of the EF experiment in order to foster adsorption by increasing the residence time of the pathogen inside the reactor; i.e., to take the effluent, T_2_, mix it with the influent solution, T_1_, and pump it back into the reactor (see Fig. 1a). In this experiment (EF-R), the treatment time (72 min), the processed volume (1 L) and the concentration of *E. coli* and *HE* employed, were the same as those in previous experiments.

The results of these runs are also shown in Fig. 2c where it can be observed that, while *E. coli* could only be observed in the influent sections of the reactor, C_1_ and e_1_, no traces of the pathogen could be detected on the AC compartment, e_2_, C_3_ and the effluent. In this way, the EF-R approach eliminates, as expected, almost all the *E. coli* in the starting contaminated solution by increasing the pathogen residence time inside the reactor.

The results for the EF-R experiments with the more resistant *HE* pathogen, also showed an increase in the efficiency of the disinfection process reaching a value close to 90%. The substantial difference in the disinfection efficiency of EF and EF-R, probably reflects the fact that by recycling, not only the residence time of the pathogen increases but also, the Fe(II) and H^+^ ions that are required for the electro-Fenton reaction are re-introduced into the system.

From the interpretation of the data at this point, it was clear that the most efficient disinfection conditions corresponded to the EF promoted generation of the •OH radical, that it was important to maintain Fe(II) and acidic conditions (∼pH3) (Sirés et al., 2014) within the system and particularly in the cathodic zone of the reactor. Therefore, in order to further improve the disinfection efficiency of the process, we defined an additional operation mode (EF-RPI) in which the recycling scheme is divided in sequential stages for which adsorption and polarization events are decoupled. In this way, sequential adsorption (15 min at a flow rate of 14 mL min^−1^) and polarization (20 min under static conditions) stages in which flow direction/polarity switching are carried out in an alternating mode, were defined and applied as shown in Table 1.

The flow rate and the time periods for each alternating stage were set from previous experiments reported by our group (García-Espinoza et al., 2019) in such a way that the retention of Fe(II), Fe(III) and acid cationic species within the reactor can be achieved. As pointed out by (Fernández et al., 2018) this is an important feature of the EF-RPI process since in the previous schemes surveyed, depletion of the Fe and acid species from the H_2_O_2_ production zone, would eventually decrease the performance of the disinfection system.

It is also important to point out that the alternating scheme of the EF-RPI operation mode not only promotes electro-Fenton generation conditions within the reactor at all times, but also pursue an scheme in which most of the AC packed bed in C_2_, works as a cathode at some point in time; thus maximizing the production of H_2_O_2_ in the presence of Fe(II) under low pH conditions (∼pH3) (Fernández et al., 2018). It is also important to note that although the alternating sequential adsorption/polarity approach, EF-RPI, takes almost twice as long (155 min) when compared to previous experiments (72 min), the time dedicated to polarization of the reactor is very similar (80 min).

The results of the set of experiments corresponding to the EF-RPI process are also shown in Fig. 2d. Inspection of the corresponding data reveals that while *E. coli* disinfection is complete in all sections of the reactor as well as in the effluent, non-viable *HE* pathogens were the only form of the pathogen that could be found. Although the distribution of non-viable *HE* in C_2_ and in a larger proportion in the effluent will probably change when several cycles are carried out, the complete inactivation of the highly resistant *HE* is with no doubt, the most important result.

**Fig. 5:**
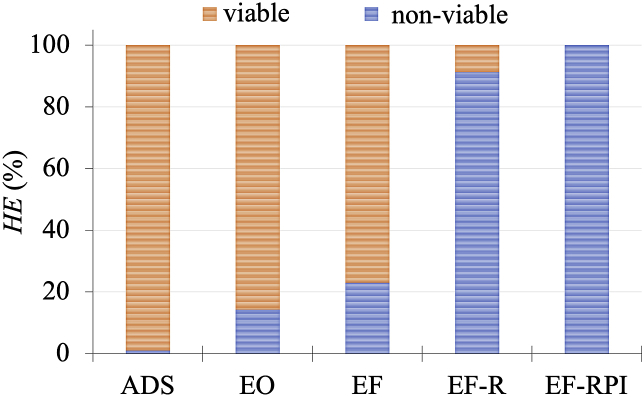
Viable and non-viable percentages of *HE* for the different operation modes surveyed.

In Fig. 5, the system’s results for integral *HE* inactivation are shown for the different operation modes surveyed. It is interesting to see that while ADS does not show any inactivation effect on the pathogen, EO and EF have modest disinfection performance; EO due to electrochemical reactions taking place at the anode and EF from direct and indirect oxidation reactions occurring in both electrodes. Further changes in the operation mode showed that while recycling the treated solution, modifies the residence time of the pathogen within the reactor and increases the adsorption and the EF induced inactivation of the *HE* pathogen in almost 65% when compared to the EF process, the introduction of a sequential flow direction/polarity switching maintains electro-Fenton generation conditions all along the C_2_ section of the reactor, thus avoiding the depletion of the Fenton mixture components and reaching full disinfection results.

### 3.4 Computer simulation of the disinfection processes under study

**Fig. 6:**
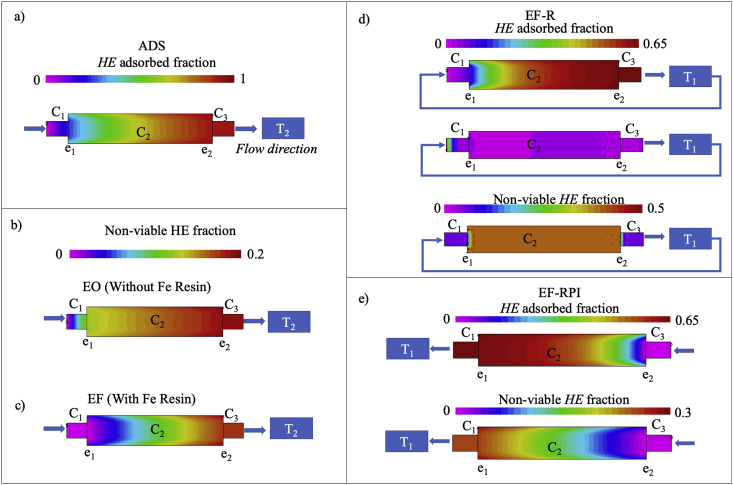
Theoretical profiles of adsorbed and non-viable *HE* along the electro-Fenton reactor under study for a) ADS, b) EO, c) EF, d) EF-R and e) EF-RPI processes.

In order to improve the understanding of the influence of the applied electric field, transport phenomena in the 3D packed bed electrode and the distribution of different chemical species along the reactor, a theoretical analysis of the *HE* disinfection processes under study was carried out using computer generated simulation. As can be seen in Fig. 6a, the simulation of *HE* distribution in the ADS process, reveals that there is an adsorption front-edge that evolves from left to right and for which the parabolic shape suggests a centered concentration profile with slight retention at the reactor’s walls.

Consistent with the experimental results, the simulation also suggests that at the end of the treatment (72 min), while there is a purple-blue zone (e_1_-C_1_ contact zone and the left hand side of C_2_ referred to simulation subdomain in Fig. 1b) in which the smaller fraction of adsorbed pollutant is located, most of the infectious material is preferentially retained in the right hand side of C_2_ and in the resin in C_3_.

When an electric potential difference is applied to promote pathogen inactivation by electrooxidation (EO), the simulation results agree well with experimental observations. The simulation field in Fig. 6b shows that in the cathodic region there is a small fraction of non-viable *HE* (probably due to the generation of the weak oxidant H_2_O_2_) and that in the anodic section of the reactor, electro-oxidation processes coupled to a relatively high density of adsorbed pathogens gives rise to a larger fraction of non-viable *HE* that results in an almost completely disinfected effluent (see Fig. 2b).

Meanwhile, when the electro-Fenton situation is considered (EF), H_2_O_2_ generated in the cathode reacts with Fe(II) ions to produce the •OH radical, which in turn promotes a rapid pathogen disinfection and an easier pollutant evacuation from the packed bed section when compared to the profile obtained for the EO process (see the difference in color scale).

In this way, the homogeneous profile of non-viable *HE* for EO shown in Fig. 6b, indicates that the inactivation of pathogens is slow and therefore, most of the infectious material is accumulated along the packed bed AC section, C_2_. When the inactivation rate is increased by the EF process (see Fig. 6c), the residence time of *HE* in the reactor is larger than the reaction rate, promoting the rapid evacuation of pollutants towards T_2_. This indicates that the inactivation process is higher for the EF approach when compared to the EO operation mode; an observation that agrees well with experimental results in which the disinfection percentage of *HE* increases in about 10%.

The importance of mixing conditions is further illustrated by the simulation results carried out for the EF-R configuration. As can be seen in Fig. 6d, recirculation of the effluent results in *HE* profiles that evolve on time towards a more homogeneous distribution of the pathogen along the reactor which in turn, is reflected by a substantially larger fraction of non-viable *HE* pathogen. This observation is in agreement with the experimental results previously described and reveal the need to promote the generation of the Fenton mixture conditions all along the system; that is, to have the electric potential, the Fe(II) and the pH conditions within the reactor so that the strong •OH radical species can be efficiently produced in the adsorbent-solution interphase in C_2_.

In this context, the simulation for the EF-RPI process was carried out and the resulting images are presented in Fig. 6e. Inspection of this computer-generated profile, shows that the *HE* adsorption and inactivation distributions, take place all along the reactor; a feature that explains the complete *HE* inactivation observed from the analytical and micrographic determination tests. As it was previously noted, this result suggests that the eggshell remnants that were found in the different reactor compartments (see Fig. 4) are the product of destruction of the *HE* protective membrane that takes place in the most efficient way when the EF-RPI approach is employed.

## 4 Conclusions

In this work, effective and complete inactivation of synthetic solutions contaminated with either, helminth egg (*HE*) or *Escherichia coli* (*E. coli*), was achieved using an electro-Fenton reactor in which adsorption and electron transfer processes simultaneously take place at the surface of polarized activated carbon (AC) particles located in the central compartment of a cylindrical reactor.

Continuous mode experiments showed that while AC adsorption (ADS) was useful by retaining *HE*, and polarization of the carbon particles (EO) resulted in 16% of pathogen inactivation, the use of an iron loaded cation exchange resin, created Fenton conditions within the reactor (EF), raising the *HE* inactivation value to 25%. Using pathogen concentration data in different parts of the reactor and a computer simulation of adsorption and of the AC potential distribution, it was possible to define a recirculation and a polarization switching operation mode (EF-RPI) that creates the conditions to not only completely disinfect *HE* contaminated water but also, to avoid the need to acidify and to add Fe(II) ions to the influent and later on, to take them away from the effluent, so that electro-Fenton reaction conditions are maintained.

Based on the high disinfection efficiency that was achieved (particularly for the highly resistant *HE* pathogen) and on the availability and cost of the materials employed to build and operate the electro-Fenton reactor under study, we believe that this approach could be an interesting contribution for the development of electrochemical disinfecting reactors, that could be an important element of human waste treatment technologies.

## CRediT authorship contribution statement

**Irma Robles:** Investigation, Visualization, Project administration, Methodology, Supervision. **Emmanuel Becerra:** Investigation, Formal analysis. **J.A. Barrios:** Investigation, Formal analysis. **C. Maya:** Investigation, Formal analysis. **B. Jiménez:** Funding acquisition. **Francisco J. Rodríguez-Valadez:** Methodology. **Fernando Rivera:** Software. **Josué D. García-Espinoza:** Investigation, Validation. **Luis A. Godínez:** Conceptualization, Writing - review & editing, Funding acquisition.

## Declaration of competing interest

None.

## Appendix A Supplementary data

Table SM-1 Electrochemical and Adsorption parameters employed for the numerical solution of equations (1), (2), (3), (4), (5). Table SM-1
