# Supplementary material for: Inactivation of helminth eggs in an electro-Fenton reactor: Towards full electrochemical disinfection of human waste using activated carbon
Source: Chemosphere. 2020 Jul;250:126260. doi: 10.1016/j.chemosphere.2020.126260 (PMC7221348; doi:10.1016/j.chemosphere.2020.126260)
Supplement: Table SM-1 — Electrochemical and Adsorption parameters employed for the numerical solution of equations (1), (2), (3), (4), (5). [file mmc1.docx]

**Table SM-1**

| ***Parameter*** | ***Value*** | ***Reference*** |
| --- | --- | --- |
| Axial dispersion coefficient, *D_ax_* (m^2^ s^-1^) | 4.8 x10^-9^ | (Wakao and Kaguei, 1983) |
| Radial dispersion coefficient, *D_r_* (m^2^ s^-1^) | 4.7 x10^-10^ | (Wakao and Kaguei, 1983) |
| Exchange current density for peroxide ECR,  i_0C_ (A m^-2^) | 6.9 | (Sarapuu et al., 2003) |
| Exchange current density for Oxygen ECR,  i_0a_ (A m^-2^) | 0.003 | - |
| Electronic transfer coefficient for both electrochemical reactions, α | 0.5 | - |
| Electrolyte conductivity, κ_elec_(S m^-1^) | 0.001 | (Fernández et al., 2018) |
| Electrode conductivity, κ_mat_(S m^-1^) | 0.007 | (Fernández et al., 2018) |
| Reaction rate (Variable Parameter) for adsorption and electro-sorption, *R_i_* (mol m^-3^ s^-1^) | $k_{ads}*C_{i}$  $k_{adspp}{*i}_{0a}exp\left( \frac{\alpha_{a}F\left( \phi_{1}-\phi_{2}-E_{ocp} \right)}{RT} \right)$*C_i_ | (Moreno, 2004) |
| k*_ads,_* AC *zone apparent adsorption constant, s^-1^* | 0.007 | - |
| Inactivation rate | $k_{in}*C_{i}$ | - |

Moreno, C.C., 2004. Adsorption of organic molecules from aqueous solutions on carbon materials. Carbon N. Y. 42, 83–94.

Sarapuu, A., Vaik, K., Schiffrin, D.J., Tammeveski, K., 2003. Electrochemical reduction of oxygen on anthraquinone-modified glassy carbon electrodes in alkaline solution. J. Electroanal. Chem. 541, 23–29. doi:10.1016/S0022-0728(02)01311-6.

Wakao, N., Kaguei, S., 1983. Heat and mass transfer in packed beds. AIChE J. Gordon and Breach Science Publishers. doi:10.1002/aic.690290627.
